# Spirooxindoles as Disruptors of Preformed Hen Egg White Lysozyme Fibrils as a Model for Neurodegenerative Diseases

**DOI:** 10.1002/cmdc.202501106

**Published:** 2026-05-11

**Authors:** Anthony Dahdah, Nilamuni H. de Silva, Ewan W. Blanch, Subashani Maniam

**Affiliations:** ^1^ School of Science STEM College RMIT University Melbourne VIC Australia

**Keywords:** alzheimer's disease, amyloid – *β*, protein misfolding, raman spectroscopy, spirooxindole, Thioflavin T assay

## Abstract

Formation of fibrils from the misfolding of proteins has been associated with being the root cause of many neurodegenerative diseases. The highly ordered structure of fibrils formed from stacked *β*‐sheet, makes the deposition of the structures and resultant development of neurodegeneration extremely difficult to treat. The importance of effective treatments against these conditions led to the highlighted study. Synthesized spirooxindole compounds labeled **Hd‐63**, **Hd‐66,** and **Hd‐74,** indicated potent abilities to perturb the structure of hen egg white lysozyme (HEWL) fibrils. The spectroscopic analyses, centered around the use of Raman spectroscopy and the changes in key protein marker bands, known as the amide I and amide III bands, show that the addition of **Hd‐63**, **Hd‐66,** and **Hd‐74** to solutions of preformed HEWL fibrils leads to morphological changes that indicate a breakdown of the highly ordered *β*‐sheet scaffold, and result in the formation of highly disordered aggregates, which do not suggest a fibril‐like nature. The formation of these disordered aggregates suggests the progression in the mechanism of fibrillation away from the saturation of fibrils in solution and instead toward the formation of off‐pathway oligomers.

## Graphical abstract

Formation of fibrils is a key factor in the development of neurodegenerative diseases. Their highly ordered structure is stabilized by strong hydrogen bonding. Synthesised spirooxindole compounds, Hd‐63, Hd‐66, and Hd‐74, disrupt fibril structure, leading to the breakdown of this order and promoting the formation of off‐pathway aggregates that lack fibrillar characteristics.

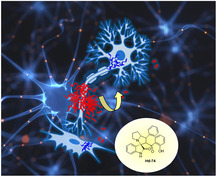

## 1 Introduction

Neurodegenerative diseases have long plagued society due to their social, physical, and economic impacts on individuals [1, 2, 3, 4]. Highlighted by conditions that result in progressive degeneration of brain tissue, neurodegenerative diseases have been investigated and studied for many years in the search for advanced and more modern treatments, as well as possible cures [5, 6, 7, 8, 9]. Parkinson's disease and Alzheimer's disease (AD) are at the forefront of global research efforts on neurodegenerative disease and are characterized as being a result of protein aggregation and inclusion body formation [10]. The aggregates generally consist of fibers containing misfolded proteins with a high *β*‐sheet content [11]. Once these fibers deposit onto brain tissue, the formation of a plaque takes place, resulting in significant attenuation of the function of the brain tissue [12, 13, 14].

A*β*_1–42_, a naturally occurring brain peptide or small protein, performs many biological roles, which include protecting the body from infections, repairing leaks in the blood–brain barrier, promoting recovery from injury, and regulating synaptic function [15]. However, when this peptide undergoes misfolding, it leads to classic morphological changes resulting in the formation of a *β*‐sheet structure. The amyloid cascade hypothesis has been at the forefront of scientific investigation into the causes of AD [16, 17, 18, 19, 20]. Misfolding of A*β*_1–42_, as a result of cleavage of the amyloidogenic region of amyloid precursor protein [21], leads to the eventual deposition of plaque onto brain tissue, resulting in the advancement of the condition. The resultant structure of the *β*‐sheet fibrils is extremely intricate and complex.

Studies carried out by Pellarin et al. [22] suggested that fibrillogenesis of a protein is mainly dependent on the stability of the amyloid‐component state. Additionally, Ma et al. proposed that the driving force of aggregation is, in fact, caused by side‐chain interactions that are predominantly hydrophobic [23]. The resultant fibrillar structure is held strongly together due to polar side‐chain interactions, which form hydrogen bonds [24].

The challenge surrounding the mechanistic understanding involved in fibril formation and inhibition has generally taken attention away from the significance of the breakdown of the highly ordered *β*‐sheet structure once fibrils have formed. Investigating the ways in which fibrils can be broken down and the protein structure altered to a less toxic state is of utmost importance. Further exploration into the mechanism involved with this process could result in the development of potent treatments that halt amyloidogenesis and improve the state of an individual who is in the late stage of neurodegenerative diseases, such as AD.

Extensive research has been carried out by various research groups investigating the changes in protein morphology using Raman spectroscopy. More specifically, changes in amide I and III bands are commonly used to characterize changes in the secondary structure since the carbonyl and amine groups of the protein backbone contribute to the hydrogen bonding for *α*‐helix, *β*‐sheet, and random coils [25, 26, 27, 28, 29].

**FIGURE 1:**
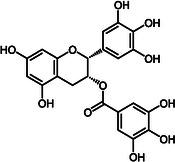
Chemical structure of EGCG.

Our current understanding of compounds that have shown potential to perturb and/or break down fibrillar structure is limited to the naturally occurring epigallocatechin gallate (EGCG) [30, 31] (Figure 1). EGCG is a green tea polyphenol, known for its antioxidant, anti‐inflammatory, and anti‐amyloidogenic properties [32, 33, 34]. EGCG has been particularly linked to the inhibition of fibril formation, as well as its ability to disassemble previously formed fibrils. Reduction in neurotoxicity takes place by interaction with monomeric, oligomeric, and fibrillar forms of A*β* [31, 35, 36].

**FIGURE 2:**
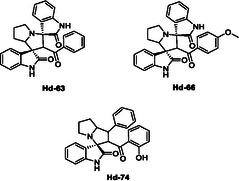
Chemical Structure of spirooxindole compounds **Hd‐63**, **Hd‐66,** and **Hd‐74**.

Previously, we have shown that spirooxindole compounds, in particular **Hd‐63**, **Hd‐66,** and **Hd‐74** (Figure 2), have inhibitory abilities against hen egg white lysozyme (HEWL) fibrillation [37, 38]. HEWL was used as a model system to study the effect on fibril structure because it is readily available, has a well‐defined structure, and is low cost [39, 40]. Like A*β*_1–42_ is made up of primarily an *α*‐helical secondary structure in its native state, while also forming highly ordered *β*‐sheet fibrils under an acidic and high‐temperature environment [41, 42].

Here, through the incorporation of Raman spectroscopy to characterize structural changes, thioflavin T (ThT) fluorescence assays to quantify the degree of fibril content in a solution, and transmission electron microscopy (TEM) for visual representation of structural changes, we have studied the ability of **Hd‐63, Hd‐66,** and **Hd‐74** to break down the structure of highly ordered, preformed HEWL fibrils.

## 2 Materials and Methods

### 2.1 Materials

HEWL and ThT were purchased from Sigma‐Aldrich and were used without further purification. Spirooxindole compounds, **Hd‐63**, **Hd‐66,** and **Hd‐74,** were synthesized by De Silva et al. as outlined in reference [43].

#### 2.1.1 Sample Preparation for Raman, ThT Fluorescence Assay, TEM, and Circular Dichroism (CD) Studies

HEWL was dissolved in Milli‐Q water at a concentration of 20 mg/mL. The solution was filtered and pH adjusted to 2.0 using 5 M HCl. HEWL solution (900 µL) was added to Eppendorf tubes, and the samples were incubated in a thermostat‐heating block at 60°C for 7 days. One sample of 20 mg/mL HEWL solution (900 µL) was kept in the refrigerator at 4°C to act as a negative control. After 7 days of incubation at 60°C, each solution of HEWL was spiked with 100 µL of synthesized spirooxindoles **Hd‐63**, **Hd‐66,** and **Hd‐74** at concentrations of 5 mM, as well as with 5 mM EGCG. **Hd‐63**, **Hd‐66,** and **Hd‐74** were dissolved using dimethyl sulfoxide (DMSO), and EGCG was dissolved using Milli‐Q water. Control studies were carried out in previous experiments to confirm that the concentrations of DMSO that exceeded 10% began to influence protein structure. Due to this, DMSO concentration was capped at 10%. Each solution was then placed in a thermostat heating block at 60°C for another 7 days. The starting point measurements for the spectroscopic analysis and imaging were done using HEWL, which had been mildly destabilized by being exposed to a pH 2.0 environment before heating.

#### 2.1.2 Raman Spectroscopic Analysis

Samples were prepared for Raman measurements by first taking a 30 µL aliquot of the mildly destabilized protein solution. After spiking the fibrillar solution with each organic compound, 30 µL aliquots were taken daily between 1 and 7 days. Samples were analyzed using a PerkinElmer station 400 Raman spectrometer, which is equipped with a 785 nm laser. The instrument could only provide accurate measurements on solid samples, so our liquid samples were prepared by the drop‐deposition technique, in which 10 µL of each sample was dried on a glass slide, which was placed in a vacuum oven to increase the rate of evaporation of the solvents. This was repeated three times until the water in the sample completely dried off, leaving the solid sample. The glass slides were covered in aluminum (Al) foil to avoid intense background signals from silica. The measurements were collected over a wavenumber range of 200–3200 cm^−1^, with the exposure time set at 3 s with 30 accumulations resulting in 90 s of data acquisition for each measurement. Measurements were completed in triplicate over three different positions of the dried sample, and the highest‐quality spectra were used for further analysis and interpretation. The Raman spectra were analyzed using MATLAB R2018b software. Data treatment that was applied to the spectra included background subtraction (“msbackadj’ function), cosmic ray removal (“hampel” function), smoothing (“sgolayfilt” function with a polynomial order of 1 and a frame length of 5, which is a Savitzky–Golay filter), and normalization, where necessary, to the intensity of the amide I region band in the spectrum (1670 cm^−1^).

#### 2.1.3 ThT Fluorescence Assay

Stock ThT solution was prepared by dissolving 8 mg of ThT in 10.0 mL of Milli‐Q water. The stock solution was filtered and stored in a refrigerator for no longer than 7 days after preparation. A dilute ThT solution was prepared by performing a 1:50 dilution of stock ThT solution in Milli‐Q water. When performing the ThT fluorescence measurements, using a 4 mL quartz fluorescence cell, 10.0 µL of sample was mixed with 4.0 mL of dilute ThT solution. Fluorescence measurements were carried out using a Fluorimax‐4 fluorescence spectrophotometer at an excitation wavelength of 440 nm and a slit width of 5 nm. Emission spectra were recorded between 440 and 700 nm with the intensities for the *λ*_max_ at 485 nm used for further interpretation.

Triplicate measurements were collected for each sample. Similar to the Raman measurements, ThT fluorescence measurements were also taken for the mildly destabilized protein, and then between 1 and 7 days after the addition of each organic compound to monitor the effect of each compound on the fibrillar structure of HEWL.

*λ*_max_ intensities at each time point for each sample were plotted in a bar graph using MATLAB R2018b software.

#### 2.1.4 TEM Imaging

Each sample (10 µL) was placed on a carbon‐coated copper grid and left in air for 20 min. Afterward, the grid was washed with Milli‐Q water and air dried. The grid was then stained with an aqueous solution of uranyl acetate (1% w/v) for 2 min. Excess stain was removed using Milli‐Q water, and the grids were then left to dry. TEM images were obtained using a JEOL 1010 transmission electron microscope operating with a 100 kV accelerating voltage.

#### 2.1.5 Circular Dichroism

HEWL solution was prepared following the same steps outlined in section 2.1.1; however, conc. sulfuric acid was used in place of 5 M HCl due to strong absorbance by chloride ions in the far ultraviolet (UV) region. Circular dichroism spectra were collected using a JASCO J‐1500 spectrometer (ATA Scientific Corporation) at 25°C using a bandwidth of 1.0 nm, a step interval of 0.1 nm, a scanning speed of 50 nm/min, a resolution of 9.1 nm, and an averaging time of 2 s. Measurements were carried out on samples, in which 20 µL of each sample was deposited onto a 0.1 cm path length quartz cuvette and then diluted with 30 µL of DMSO. Measurements were taken in the far UV (190–260 nm) region. Triplicate scans were measured and averaged. Raw data were then processed using MATLAB R2018b software. Processing of the CD data involved smoothing the data using the *“*sgolayfilt” function with a polynomial order of 1 and a frame length of set. This function incorporated the use of the Savitsky–Golay filter to increase the precision of the dataset.

## 3 Results and Discussion

The ability of **Hd‐63**, **Hd‐66,** and **Hd‐74** to perturb the highly ordered structure of HEWL fibrils was analyzed using Raman and fluorescence spectroscopy, circular dichroism, and TEM. EGCG was included in the experiment to compare the effectiveness of the synthesized spirooxindole compounds.

### 3.1 Raman

**FIGURE 3:**
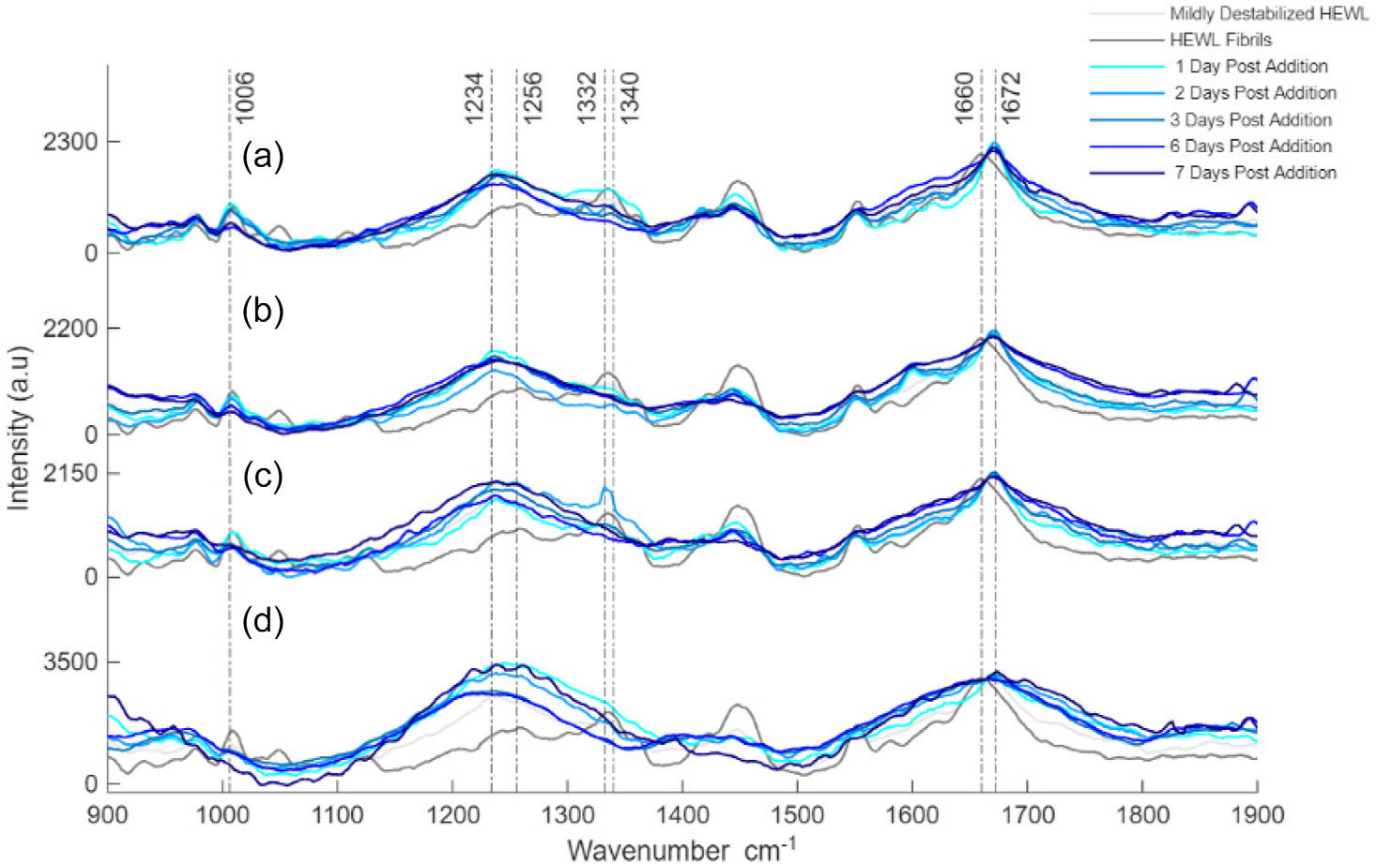
Raman spectra depicting the effect of spirooxindole compounds (a) **Hd‐63**, (b) **Hd‐66**, (c) **Hd‐74** and organic compound, and (d) EGCG on the structure of HEWL fibrils.

Raman spectroscopy was used to characterize the morphological changes of HEWL in the presence of the spirooxindole compounds **Hd‐63**, **Hd‐66**, **Hd‐74,** and EGCG. Structural changes of HEWL can be observed by analyzing variations in the amide I and III regions. HEWL fibrils are made up of a primarily highly ordered *β*‐sheet structure [40]. From the Raman spectra (Figure 3), this structure is characterized by classic amide I and III regions bands, with the amide I region band for native HEWL being positioned at 1660 cm^−1^ and HEWL fibrils resulting in an amide I region band positioned at 1672 cm^−1^. The relative positions of these bands are caused by the secondary structure composition of each morphology of HEWL. Native HEWL, which is primarily made up of *α*‐helix [44], classically results in amide I regions bands at ∼1660 cm^−1^ and HEWL fibrils, which are primarily made up of *β*‐sheet, result in a band at ∼1670 cm^−1^. Furthermore, prominent presence of *α*‐helix, as expected from the native HEWL sample, results in amide III region bands positioned at 1260–1340 cm^−1^, and *β*‐sheet prominent structures result in an amide III band positioned at 1230–1245 cm^−1^ [45, 46, 47].

Observation of the Raman spectra for each sample measured indicates that the addition of each compound to a solution of HEWL fibrils does not result in an overall shift in the position of the amide I and III region bands; however, their addition results in significant broadening of the bands. Broadening of amide bands in Raman spectra has been documented to be related to protein morphology described as disordered aggregates or random coil caused by the disorder of *β*‐sheet structure [48, 49, 50, 51]. The broadening of bands was observed upon the exposure of the protein to higher temperature conditions along with a low pH environment [52]. This suggests that the addition and continual incubation of HEWL fibrils in the presence of 5 mM **Hd‐63**, **Hd‐66**, **Hd‐74,** and EGCG may not completely remove the presence of *β*‐sheet in the secondary structure of HEWL but perturb the *β*‐sheet structure, significantly decreasing the overall *β*‐sheet content of the protein.

Comparison of the Raman spectra collected for each sample over the course of the experiment indicates clear differences in the band identities in the amide I and III regions. Significant broadening of both the amide I and III regions takes place when HEWL fibrils, which present relatively sharp bands, are exposed to anti‐amyloidogenic compounds, resulting in relatively broader bands. Exposure of HEWL fibrils to the compounds **Hd‐63**, **Hd‐66**, **Hd‐74,** and EGCG does not result in the structure of HEWL returning to its native state, indicating that the process of fibrillation is not reversible in this situation. The overall structure of HEWL, 7 days after the addition of each compound, suggests an off‐pathway highly disordered oligomeric aggregate, with little to no structural order. This is evident based on the significant broadening of the amide I region band, along with the complete loss of structural integrity presented by the amide III band, which can be initially observed for both native and fibrillar HEWL.

Semiquantitative analysis of the Raman spectra supports the suggestion that the addition of each of the compounds results in the formation of a less ordered structure of HEWL. The broadening of the amide I region band profile with continued incubation of HEWL fibrils in the presence of each compound can be further investigated using full width half maximum (FWHM) calculations. FWHM values (Table 1) are semiquantitative observational values used to describe the relative order of a structure. Raman spectroscopy is extremely sensitive to the structural order of a sample; therefore, incorporating FWHM values can act as a powerful tool to describe morphological changes taking place when samples are exposed to varying experimental conditions. An increase in relative broadness or a decrease in structural disorder can be defined by an increase in the FWHM value.

**TABLE 1:** Time‐dependent full‐width half maximum height (cm^−1^) measurements for amide I band intensity of Raman spectra in Figure 3.

Measurement	Hd‐63	Hd‐66	Hd‐74	EGCG
Mildly destabilized HEWL	52	52	52	52
HEWL fibrils	36	36	36	36
1 day post addition	18	34	38	120
2 days post addition	30	36	52	180
3 days post addition	34	60	60	186
6 days post addition	126	162	148	154
7 days post addition	62	172	174	194

Upon addition of 5 mM **Hd‐63**, **Hd‐66,** and **Hd‐74** to a solution of preformed fibrils, the overall order of the structure of the fibrils is relatively unaffected. The relative order of the fibrillar structure when exposed to **Hd‐63** initially increases based on the decrease in the FWHM value. After 2 days of incubation of the solution in the presence of **Hd‐63** and **Hd‐66**, the structure remains unaffected, with a relative change taking place only in the presence of **Hd‐63.** This indicates a slight loss of structural order compared to the previous measurement; however, the FWHM value remains close to the initial fibrillar value. Interestingly, 5 mM **Hd‐74** can be seen to begin to have an effect on the structure of the fibrils, resulting in an initial broadening of the amide I band and is highlighted by an increase in the FWHM value to 52. Additionally, it is 3 days after its addition that **Hd‐66** begins to result in disordering the structure of HEWL, and 6 days after the addition of **Hd‐63** that a change can be observed. **Hd‐74,** on the other hand, results in a progressive change, which can be seen by a continual increase in the FWHM values over the course of the experiment.

Interestingly, 7 days after the addition of **Hd‐63**, it appears that the fibrillar structure has been able to overcome the effect of the inhibitor compound and reorder its secondary structure, resulting in an unexpected increase in structural integrity. It is important to note that significant changes to the structure of the fibrils appear to take place between 3 and 6 days of incubation. Further detailed experimentation will aid in better understanding the mechanistic changes taking place.

The addition of EGCG, as discussed earlier, was to act as a reference compound. EGCG has long been studied for its medicinal properties, and from the data collected in the Raman spectra, it can be clearly observed that its addition to a solution of HEWL fibrils results in significant attenuation of the protein secondary structure with a substantial decrease in relative structural order observed even 1 day after its addition. With continued incubation in the presence of EGCG, an increase in the amide I region band broadening can be observed and is highlighted by the FWHM value reaching 194 after 7 days.

The trends in the Raman data observed suggest that the compounds **Hd‐63**, **Hd‐66,** and **Hd‐74** result in significant morphological changes when added to a solution of HEWL fibrils. The application of these compounds can be compared to EGCG since the broadening of the amide I region band is evident upon incubation of the solution postspiking.

### 3.2 Fluorescence

**FIGURE 4:**
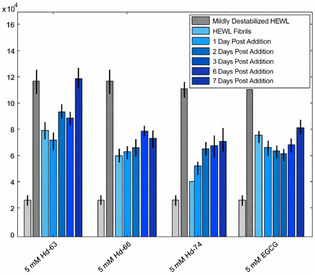
ThT fluorescence spectra displaying the changes in relative fluorescence intensity for samples of HEWL fibrils, which had been spiked with 5 mM **Hd‐63**, **Hd‐66**, **Hd‐74,** and EGCG over the course of 7 days. The standard error for triplicate measurements was determined to be ± 5%.

The ability of **Hd‐63**, **Hd‐66,** and **Hd‐74** to perturb the structure of HEWL fibrils was also analyzed by incorporating the use of a ThT fluorescence assay. The breakdown of HEWL fibrils was monitored over the course of 7 days after spiking each solution with 5 mM of **Hd‐63**, **Hd‐66**, **Hd‐74,** and EGCG (Figure 4). Changes in the fluorescence intensity can be clearly observed and indicate that there are signs of structural changes taking place, which affect the overall fibril content in the solution being measured. After the addition of 5 mM **Hd‐63**, a sudden ∼30% decrease in fluorescence intensity can be observed, which continues to decrease up to 2 days, resulting in a 37% drop‐off in overall fluorescence intensity. However, after 3 days of incubation of HEWL fibrils in the presence of 5 mM **Hd‐63** until the end of the experiment, an increase in fluorescence intensity is observed, with the relative intensity 7 days after the addition of **Hd‐63** being similar to the initial fibril measurement. This observation suggests, similar to the Raman observations, that **Hd‐63** does not have a significant effect on perturbing the structure of HEWL fibrils permanently, and its addition to the solution, although it may initially compromise the structure of HEWL fibrils, ultimately suggests that the fibrillar nature is resilient enough to overcome its presence.

On the other hand, the addition of **Hd‐66** and **Hd‐74** indicates a significant loss of the fibrillar presence within the solution. Upon initial addition of both compounds, a significant decrease in fluorescence intensity can be observed, with **Hd‐74** being the more potent compound. This is then followed by steady and slight increases in intensity, which suggest that the fibril structure is attempting to overcome the effects of these compounds. By the completion of the experiment, the compounds have a significant effect on the overall content of fibrils within the solution, suggesting that they can perturb the fibrillar structure and retard any further growth in vitro. The overall decreases in fibrillar concentration after the addition of 5 mM **Hd‐66** and **Hd‐74** are 35% and 37%, respectively. Interestingly, the data collected and analyzed for the sample containing EGCG also result in similar observations that a decrease in fluorescence intensity is clearly observed, suggesting that the fibrillar structure is being broken down. However, the overall fluorescence intensity at the completion of the experiment is slightly higher than both the **Hd‐66‐** and **Hd‐74‐**containing samples, with an overall decrease in fibrillar concentration being 29%. This discovery suggests that both **Hd‐66** and **Hd‐74** could be more potent agents for the breakdown of fibrils as compared to the commonly studied EGCG.

### 3.3 TEM

TEM is performed to analyze the samples visually to observe the morphological changes, which take place when HEWL fibrils are exposed to a series of compounds. The compounds include **Hd‐63**, **Hd‐66**, **Hd‐74,** and EGCG. Significant changes in the morphology of HEWL can be observed, first being that the initial mildly destabilized state of the protein appears as clumps of disordered aggregates varying in distribution. Upon applying denaturing conditions, HEWL fibrils appear as a complex network of structurally ordered fibers, which overlap with each other.

**FIGURE 5:**
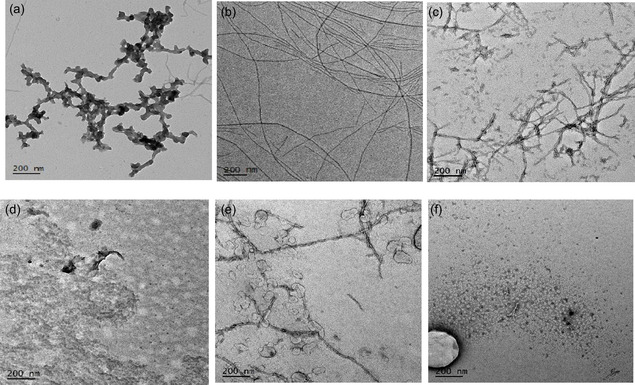
TEM images for (a) mildly destabilized HEWL, (b) HEWL fibrils, and HEWL fibrils incubated in the presence of 5 mM (c) **Hd‐63**, (d) **Hd‐66**, (e) **Hd‐74**, and (f) EGCG for 7 days.

Once the fibrils are exposed to each of the compounds, clear changes in the structure can be observed (Figure 5). Firstly, the addition of **Hd‐63** results in the formation of aggregated clumps, as well as a significant decrease in the ordered structure being visually present. Furthermore, the structure can also be identified as more spaghetti‐like with strands that appear much shorter in their overall length.

The addition of **Hd‐66**, **Hd‐74,** and EGCG suggests that the overall structural integrity of the protein is completely lost after spiking the fibril solution, with clumps of aggregates forming and being randomly distributed in space. Of the three, **Hd‐74** suggests some signs of fibrils being present; however, the overall structure appears to be dominated by disordered structure, which has resulted in disordered aggregates being formed. The hairlike structures that can be observed are surrounded by clumpy aggregates, are relatively shorter in length, and appear disordered based on their relative broadening. The TEM images indicate that the addition of 5 mM **Hd‐63**, **Hd‐66**, **Hd‐74,** and EGCG results in the breakdown of the fibrillar structure, resulting in the formation of disordered aggregates.

### 3.4 Circular Dichroism

Circular dichroism (CD) was included in this study to act as a confirmatory technique for the conclusions made from the Raman data regarding the secondary structure of HEWL fibrils after being exposed to spirooxindole compounds **Hd‐63**, **Hd‐66,** and **Hd‐74** (see Supporting Information, Figures S1–S3). Similar to Raman spectroscopy, CD spectra are also sensitive to secondary structure morphology with *α*‐helical dominant proteins resulting in bands at ∼208 and 222 nm [53] and *β*‐sheet dominant proteins resulting in bands at ∼190 nm and a negative shoulder at ∼215–220 nm [54, 55]. As discussed with the Raman data, analysis of the CD spectra indicates significant broadening of the characteristic marker bands, indicating a loss of structural integrity and a relative increase in structural disorder.

Upon analyzing the CD spectra (Figures S1–S3), the classical changes can be observed when mildly destabilized HEWL morphologically changes to form the fibrils with a clear decrease in overall intensity of the 192 nm band. This occurs after applying the mild denaturing conditions of pH 2.0 environment along with incubation at 60°C for 7 days. Upon introducing the spirooxindole compounds to the solutions of HEWL fibrils, clear differences in the spectra can be observed, which suggest that the structure after 7 days changes from fibrils to something with a relatively low degree of structural order, highlighted by the extreme broadening of the characteristic bands that appear in the spectra and are normally used to identify varying secondary structure morphologies. As discussed in previous studies, these bands are positioned at ∼194, ∼208, and ∼220 nm [56, 57]. The broadening of the characteristic marker bands indicates the relative decrease in structural order emphasized by the morphological changes taking place, as the highly ordered structure of the *β*‐sheet fibrils is broken down to off‐pathway disordered aggregates of HEWL.

## 4 Conclusion

To summarize, the spectral measurements upon the addition of our novel compounds **Hd‐63**, **Hd‐66,** and **Hd‐74** suggest that significant morphological changes take place with a clear indication that the formation of a highly disordered structure dominates. This is highlighted by the increase in relative amide I region band broadness, which can be semi‐quantified using the FWHM values obtained from the Raman data. Overall, the incubation of HEWL fibrils in the presence of the spirooxindole compounds **Hd‐63**, **Hd‐66,** and **Hd‐74** results in clear changes to the secondary structure, resulting in a breakdown of the highly ordered fibrillar structure into a highly disordered structure, which is most likely a disordered aggregate. Of the three compounds, **Hd‐74** presents the most efficient ability to break down the structure of fibrils, followed by **Hd‐66,** which also indicates prominence in perturbing the structure of HEWL fibrils, although taking slightly longer to have an initial effect. The effect of **Hd‐63** can be observed after 6 days of being added to the solution of HEWL fibrils, but then appears to have significantly less effect toward the end of the experiment. Additionally, the relatively lower fluorescence intensities observed for the samples that were spiked with **Hd‐66** and **Hd‐74** indicate the decreased concentration of fibrillar species in solution.

The visual observations of the TEM images indicate the formation of a structure, which appears to be amorphous and clumpy in nature, concluding that the addition of these compounds does not result in the fibrillar structure remaining intact, instead undergoing a high degree of change away from the traditional mechanistic expectation of an increase in structural order and more toward an off‐pathway highly disordered oligomeric species.

Finally, the combination of the data suggests that the addition of each of the spirooxindole compounds, **Hd‐63**, **Hd‐66,** and **Hd‐74** displays significant potential to perturb the highly ordered structure of *β*‐sheet fibrils, which could result in a possible treatment option for individuals suffering from AD and other neurodegenerative diseases.

## Supporting Information

Additional supporting information can be found online in the Supporting Information section.

## Author Contributions

The manuscript was written through contributions of all authors.

## Funding

This study was supported by RMIT University.

## Conflicts of Interest

The authors declare no conflicts of interest.

## Supporting information

Supplementary Material

## Data Availability

The data that support the findings of this study are available from the corresponding author upon reasonable request.
