# Supplementary material for: Spirooxindoles as Disruptors of Preformed Hen Egg White Lysozyme Fibrils as a Model for Neurodegenerative Diseases
Source: ChemMedChem. 2026 May 11;21(9):e202501106. doi: 10.1002/cmdc.202501106 (PMC13160252; doi:10.1002/cmdc.202501106)
Supplement: Supplementary file 1 — Supplementary Material [file CMDC-21-e202501106-s001.pdf]

RMIT Classification: Trusted

Supporting Information

Spirooxindoles as Disruptors of Pre-formed Hen Egg White Lysozyme Fibrils as a Model in Neurodegenerative Diseases

Anthony Dahdah, Nilamuni H. de Silva, Subashani Maniam, Ewan W. Blanch

School of Science, STEM College, RMIT University, 124 La Trobe Street, Melbourne, VIC, 3001, Australia

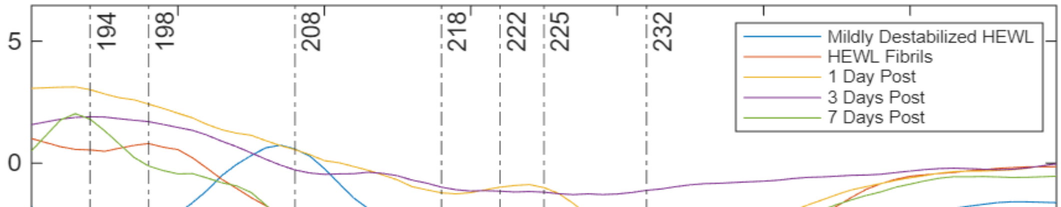

RMIT Classification: Trusted

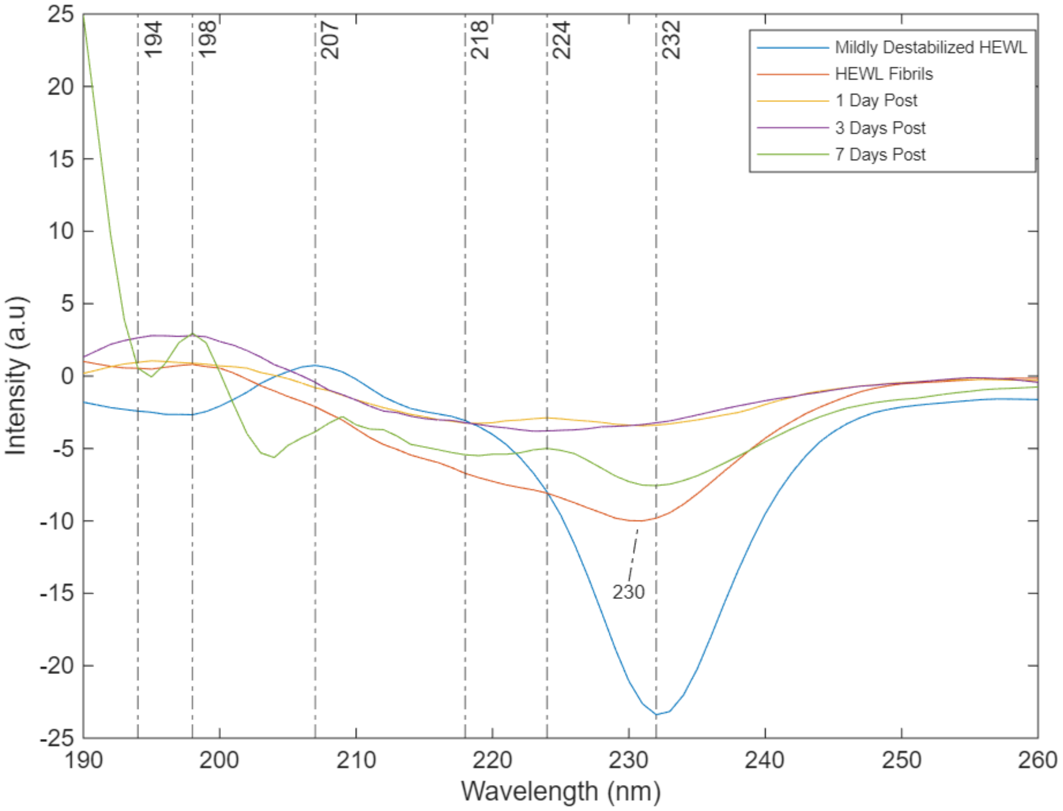

Figure S2. Circular dichroism spectra depicting the effect of 5 mM **Hd-66** against the structure of HEWL fibrils.

RMIT Classification: Trusted

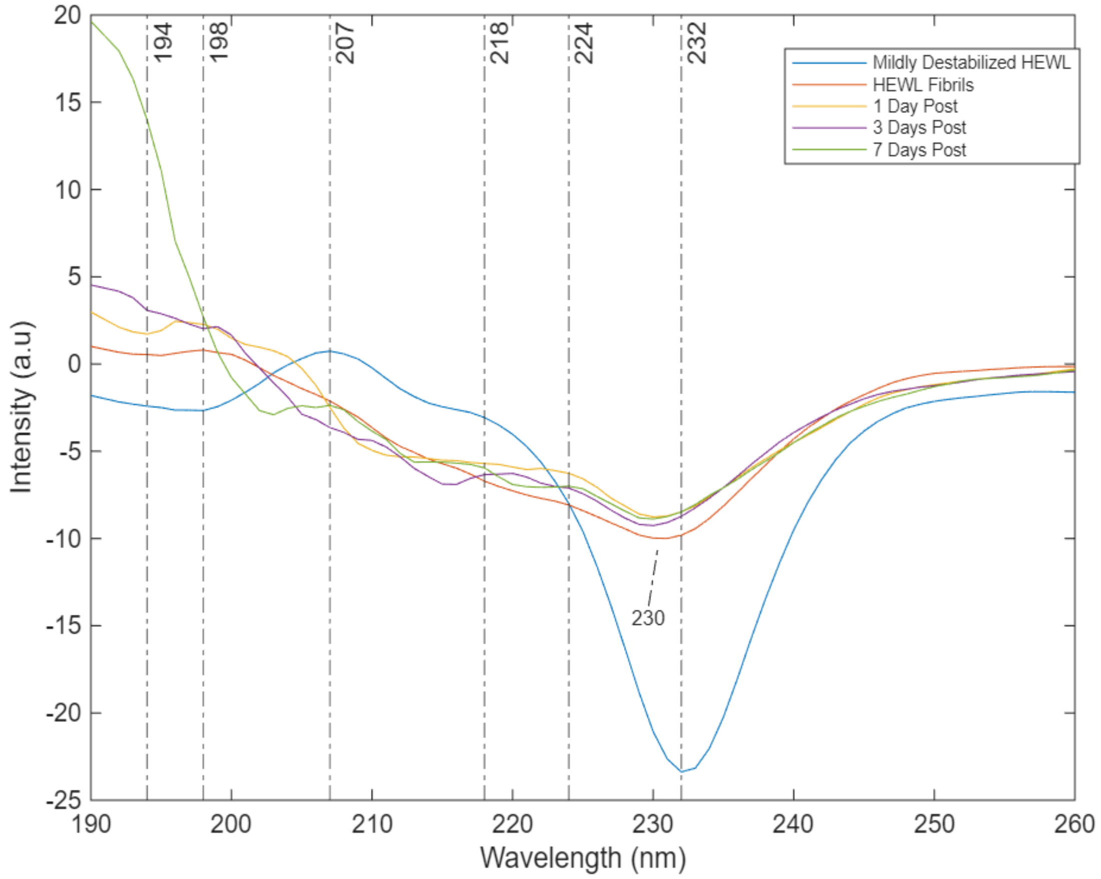

Figure S3. Circular dichroism spectra depicting the effect of 5 mM **Hd-74** against the structure of HEWL fibrils.
